# The Identification and Characterization of a Novel Alginate Lyase from *Mesonia hitae* R32 Exhibiting High Thermal Stability and Potent Antioxidant Oligosaccharide Production

**DOI:** 10.3390/md23040176

**Published:** 2025-04-17

**Authors:** Yongshang Ye, Zhiyu Li, Ying Zhou, Xiujun Gao, Dingfan Yan

**Affiliations:** Department of Biotechnology, School of Marine Science and Technology, Harbin Institute of Technology, Weihai 264209, China; yeyongshang2022hit@163.com (Y.Y.); 18561859926@163.com (Z.L.); 17737879056@163.com (D.Y.)

**Keywords:** alginate lyase, alginate oligosaccharides, PL6 family, enzymatic properties, antioxidant activity

## Abstract

Alginate lyases are of great importance in biotechnological and industrial processes, yet research on these enzymes from *Mesonia* genus bacteria is still limited. In this study, a novel PL6 family alginate lyase, MhAly6, was cloned and characterized from the deep-sea bacterium *Mesonia hitae* R32. The enzyme, composed of 797 amino acids, contains both PL6 and GH28 catalytic domains. A phylogenetic analysis revealed its classification into subfamily 1 of the PL6 family. MhAly6 showed optimal activity at 45 °C and pH 9.0, retaining over 50% activity after 210 min of incubation at 40 °C, highlighting its remarkable thermal stability. The enzyme exhibited degradation activity toward sodium alginate, Poly M, and Poly G, with the highest affinity for its natural substrate, sodium alginate, producing alginate oligosaccharides (AOSs) with degrees of polymerization (DP) ranging from 2 to 7. Molecular docking identified conserved catalytic sites (Lys241/Arg262) and Ca^2+^ binding sites (Asn202/Glu234/Glu236), while the linker and GH28 domain played an auxiliary role in substrate binding. Antioxidant assays revealed that the MhAly6-derived AOSs showed potent radical-scavenging activity, achieving 80.64% and 95.39% inhibition rates against DPPH and ABTS radicals, respectively. This work not only expands our understanding of alginate lyases from the *Mesonia* genus but also highlights their biotechnological potential for producing functional AOSs with antioxidant properties, opening new avenues for their applications in food and pharmaceuticals.

## 1. Introduction

Alginic acid is an acidic linear polysaccharide composed of *β*-D-mannuronic acid (M) and *α*-L-guluronic acid (G), linked by *β*-1,4-glycosidic bonds. It is widely found in the cell walls and intracellular matrices of brown algae (e.g., *Laminaria* and *Macrocystis*) [1]. Based on the composition of sugar units, alginic acid can be classified into poly *β*-D-mannuronic acid (Poly M), poly *α*-L-guluronic acid (Poly G), or heteropolymers. Sodium alginate, as a hydrocolloid, has broad applications in biomedicine [2], wastewater purification [3], and soil improvement [4,5]. However, its poor solubility and bioavailability limit its utilization. Alginate oligosaccharides (AOSs), small-molecule products derived from the hydrolysis of alginate with degrees of polymerization (DPs) typically ranging from 2 to 25 [1], exhibit superior solubility and broader application potential due to their lower molecular weight (Mw). Recently, AOSs have attracted significant attention for their diverse biological functions, including antioxidant [6], antitumor [7], immunomodulatory [8,9], and plant growth-regulating activities [10,11], highlighting their value in biomedical research [12,13] and agriculture [14,15].

The *β*-1,4-glycosidic bonds in sodium alginate can be cleaved via physical, chemical, or enzymatic methods to produce AOSs. Physical [15,16] and chemical approaches [17] face challenges such as high equipment costs, operational risks, and byproduct pollution. In contrast, enzymatic hydrolysis offers advantages of high efficiency, mild condition, and low energy consumption [18,19]. During enzymatic degradation, alginate lyases catalyze *β*-elimination reactions to break *β*-1,4-glycosidic bonds, generating monosaccharides or oligosaccharides. Based on the substrate specificity, alginate lyases are categorized into three groups: Poly M lyases (EC 4.2.2.3), Poly G lyases (EC 4.2.2.11), and bifunctional enzymes capable of degrading both Poly M and Poly G. According to the CAZy database, alginate lyases are classified into 16 polysaccharide lyase (PL) families (PL5, 6, 7, 8, 14, 15, 17, 18, 31, 32, 34, 36, 38, 39, 41, and 44) based on the sequence homology of the catalytic domain [20]. Endolytic alginate lyases randomly cleave internal glycosidic bonds to produce low-DP unsaturated AOSs, while exolytic enzymes generate unsaturated monosaccharides. The currently characterized alginate lyases are primarily derived from marine microorganisms, such as *Vibrio* [21,22], *Microbulbifer* [23], *Flavobacterium* [24], and *Streptomyces* [25,26]. The genus *Mesonia*, first reported by Nedashkovskaya et al. in 2003 [27], comprises 11 validly published species (https://lpsn.dsmz.de/genus/mesonia, accessed on 26 February 2025) [28]. However, research on *Mesonia* remains largely focused on novel species identification, with limited studies on functional proteins. Numerous genes in sequenced *Mesonia* genomes remain uncharacterized [29].

In our previous work, the bacterial strain *M*. *hitae* R32 was isolated from a depth of 150 m in the South Atlantic Ocean (13.36° W, 15.17° S), demonstrating growth capability with sodium alginate as the sole carbon source. However, the enzymatic properties and degradation mechanisms of its alginate lyase remain unelucidated [30]. In this study, a novel PL6 family alginate lyase, MhAly6, from *M*. *hitae* R32 was cloned and expressed. The enzyme exhibited excellent thermal stability and tolerance to metal ions, effectively degrading sodium alginate to produce AOSs with a DP ranging from 2 to 7. These AOSs showed significant antioxidant activity.

## 2. Results and Discussion

### 2.1. Analysis of Gene and Protein Sequence of MhAly6

**Figure 1:**
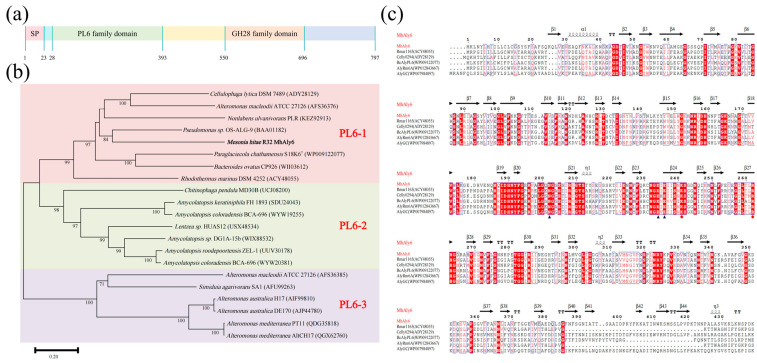
Comprehensive characterization of MhAly6 using multiple tools. (**a**) SP: signal peptide (1–23), PL6 family domain (28–393), GH28 family domain (550–696). (**b**) Phylogenetic analysis of MhAly6 and other alginate lyases of the PL6 family. Subfamilies 1, 2, and 3 are highlighted with pink, green, and purple shading, respectively, and their names are labeled in red text. (**c**) Multiple sequence alignment analysis of MhAly6 with selected alginate lyases of the PL6 family (partial sequences). Catalytic sites and Ca^2+^ binding sites are marked with red circles and blue triangles, respectively.

The open reading frame of the *MhAly6* gene spans 2391 bp, encoding a protein consisting of 797 amino acid residues. The predicted structure of MhAly6 includes an N-terminal signal peptide (Met1-Ser23, 23 amino acids), a PL6 family domain (Val28-Ala393), and a GH28 family domain (Pro550-Ile696) (Figure 1a). Notably, MhAly6 is distinguished from previously reported alginate lyases by the presence of a GH28 family domain. According to the CAZy database, proteins in the GH28 family are predominantly polygalacturonases (pectinases) [31]; however, no pectin-degrading activity was detected for MhAly6 in this study. Through a multiple sequence alignment with previously characterized GH28 family polygalacturonases, it was observed that MhALY6 retains two of the three conserved catalytic residues (Asp519 and Asp520), but exhibits a Lys substitution at position 498 instead of the conserved catalytic residue Asp (Figure S1). We hypothesize that the absence of Asp498 might be responsible for MhALY6’s inability to degrade pectin. Interestingly, these three catalytic residues are located outside the predicted GH28 family domain (Pro550-Ile696), which shows partial discrepancy with the annotation in the NCBI Conserved Domain Database. In our ongoing study, we plan to employ site-directed mutagenesis to substitute Lys498 with Asp, aiming to investigate its potential impact on the pectin-degrading capability and further elucidate the structural characteristics of the GH28 family domain. The theoretical Mw of mature MhAly6 is approximately 86.70 kDa, with an isoelectronic point of 7.58 and an instability index (II) of 28.26, indicating that it can be classified as a stable protein. The predictions from the SoluProt server suggested that MhAly6 could be expressed in soluble form in *Escherichia coli*, which was experimentally validated in subsequent studies. The NCBI sequence alignment revealed that MhAly6 shares the highest similarity (84.65%) with an uncharacterized PL6 family protein from *Salegentibacter echinorum* (WP083572075). The phylogenetic analysis placed MhAly6 within subfamily 1 of the PL6 family (Figure 1b). Its sequence similarities to characterized enzymes were as follows: 56.34% with Celly0294 from *Cellulophaga lytica* DSM 7489 [32]; 53.23% with AlyGC from *Glaciecola chathamensis* S18K6^T^ [33]; 50.41% with BcAlyPL6 from *Bacteroides clarus* YIT 12,056 [34]; 40.26% with Rmar1165 from *Rhodothermus marinus* DSM 4252 [32]; and 40.26% with AlyRm6A from *R. marinus* 4252 [35]. The combined conserved domain analysis and multiple sequence alignment identified Lys241 and Arg262 as the Brønsted base and acid, respectively, participating in the catalysis. Additionally, Asn202, Glu234, and Glu236 were proposed as Ca^2+^ binding sites. These residues were subsequently validated by molecular docking and are highly conserved in the characterized alginate lyases of the PL6 family (Figure 1c). Overall, MhAly6 has been identified as a novel dual domain alginate lyase of the PL6 family.

### 2.2. Purification and Biochemical Properties of MhAly6

**Figure 2:**
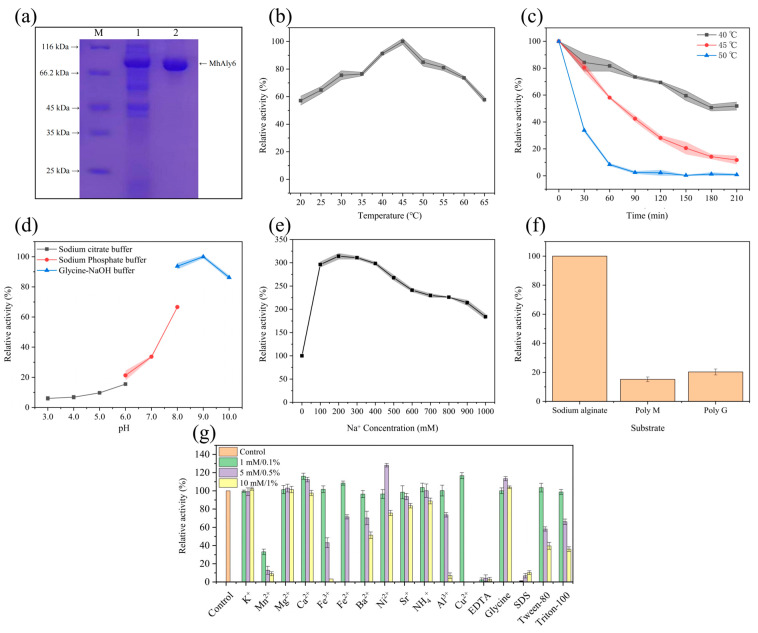
Biochemical characterization of MhAly6. (**a**) Lane M: protein marker, Lane 1: MhAly6 before purification, Lane 2: MhAly6 after purification. (**b**) Relative activity of MhAly6 at different temperatures (20–65 °C). (**c**) Thermal stability of MhAly6 at various temperatures (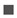: 40 °C, 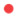: 45 °C, 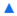: 50 °C). (**d**) Relative activity of MhAly6 at different pH values (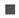: sodium citrate buffer for pH 3.0–6.0, 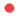: sodium phosphate buffer for pH 6.0–8.0, 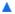: glycine-NaOH buffer for pH 8.0–10.0). (**e**) Relative activity of MhAly6 at varying Na^+^ concentrations (0–1000 mM). (**f**) Substrate specificity of MhAly6 toward sodium alginate, Poly M, and Poly G. (**g**) Effects of metal ions and chemical reagents on the activity of MhAly6. (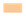: control, 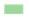: low concentration (1 mM/0.1%), 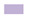: moderate concentration (5 mM/0.1%), 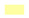: high concentration (10 mM/0.1%)).

The full-length MhAly6 gene was successfully cloned and expressed in *E. coli* BL21(DE3). The purification results revealed a single clear band between 66.2 kDa and 116.0 kDa, consistent with the theoretical Mw of the MhAly6 (86.70 kDa) (Figure 2a).

MhAly6 exhibited maximum activity at its optimal temperature of 45 °C, retaining over 80% activity at 35 °C, 50 °C, and 55 °C (Figure 2b). After incubation for 60 min at 40 °C, approximately 80% activity remained, and over 50% activity was retained even after 210 min. However, the activity decreased to 60% and 10% after incubation at 45 °C and 50 °C after 60 min, with 90% and 100% loss observed after 210 min, respectively (Figure 2c). As shown in Table 1, most reported alginate lyases of the PL6 family exhibit optimal temperatures between 30 and 45 °C. For instance, TsAly6A, ALFA4, AlyM2, AlgL6, AlyRm1, and TAPL6 show maximum activity below 40 °C, while AlyPL6 shares the same optimal temperature (45 °C) as MhAly6. VpAly-VII and AlyRmA exhibit even higher optimal temperatures (>45 °C). Notably, MhAly6 demonstrated a superior thermal stability compared to that of most enzymes, retaining more than 50% activity after incubation at 40 °C for 210 min, whereas others lost more than 50% activity within 60 min under similar conditions. Previous studies have suggested that the proportion of proline and arginine residues correlates with thermal stability. The rigid cyclic side chain of proline restricts the backbone conformational flexibility [36], while the highly protonated guanidinium group of arginine facilitates diverse electrostatic interactions at physiological pH [37]. AlyRmA, with the highest reported thermal stability among the PL6 family enzymes listed in Table 1, contains 6.88% proline and 9.12% arginine. In contrast, MhAly6 has only 3.39% and 2.26% of these residues. This implies that enhancing the proline/arginine content via amino acid substitution could further improve the thermal stability of MhAly6. As for the optimal pH, the MhAly6 activity increased under alkaline conditions, with peak activity observed at pH 9.0, and retaining 86.19% residual activity at pH 10.0 (Figure 2d). Considering that alkaline pretreatment is commonly employed in the extraction of alginate from brown algae, the alkaliphilic properties of the MhAly6 are well suited for industrial applications.

Consistent with most alginate lyases, MhAly6 displayed Na^+^-dependent activation (Figure 2e). The enzyme activity increased with the Na^+^ concentration, reaching 3.14-fold of the control at 200 mM. Even at Na^+^ concentrations ranging from 300 to 900 mM, the activity remained over twice the control level. Substrate specificity assays revealed that MhAly6 had relative activities of 100%, 15.20%, and 20.28% for sodium alginate, Poly M, and Poly G, respectively (Figure 2f). This indicated a strong preference for natural sodium alginate over homopolymeric substrates.

The effects of various metal ions and chemicals on MhAly6 were also investigated (Figure 2g). The enzyme activity was enhanced by Ca^2^⁺, Mg^2^⁺, and glycine at all tested concentrations (1–10 mM or 0.1–1%). In contrast, Mn^2^⁺, EDTA, and SDS strongly inhibited the activity at low concentrations (1 mM/0.1%). Specifically, EDTA binds to the divalent metal ions in MhAly6 (e.g., Ca^2^⁺), forming stable chelates that remove the metal ions from the enzyme, ultimately leading to its deactivation. SDS could disrupt the hydrophobic interactions critical for maintaining the tertiary structure of the enzyme. This made the enzyme lose its native conformation, and the spatial structure of the active site was disrupted, preventing substrate binding or catalysis. At higher concentrations (5–10 mM/0.5–1%), most of the tested metal ions and chemical reagents reduced the enzyme activity. However, Ni^2^⁺ showed a unique trend. It inhibited the activity at a low concentration (1 mM), enhanced the activity at a moderate concentration (5 mM), and then inhibited it again at a high concentration (10 mM). In industrial processes for preparing AOSs from brown algae, various metal ions are inevitably introduced. MhAly6’s tolerance to common industrial metal ions highlights its significant practical potential.

In summary, although MhAly6 did not surpass all reported enzymes of the PL6 family in every aspect, its exceptional thermal stability, tolerance to metal ions, and strong affinity for natural alginate make it a highly promising candidate for degrading complex brown algal biomass.

### 2.3. Analysis of the Degradation Products of MhAly6

**Figure 3:**
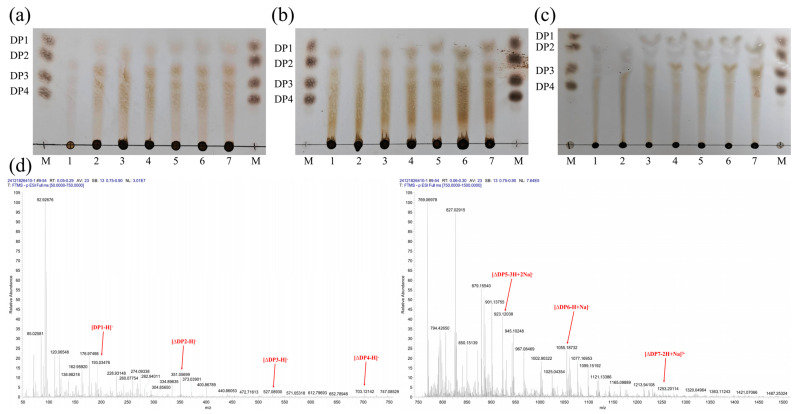
Analysis of the degradation products of MhAly6. (**a**–**c**) TLC analysis of the degradation products from sodium alginate, Poly M, and Poly G, respectively. M: DP 1–4 standards; lanes 1–7: Degradation products of the three substrates at reaction times of 0.5 h, 1 h, 2 h, 3 h, 6 h, 12 h, and 24 h using purified MhAly6. (**d**) ESI-MS analysis of the sodium alginate degradation products after 24 h.

The degradation products of MhAly6 acting on three substrates (sodium alginate, Poly M, and Poly G) at different time points were analyzed using thin-layer chromatography (TLC). The results (Figure 3a,b) revealed similar product profiles for sodium alginate and Poly M, with monosaccharides detected within 30 min of degradation. In contrast, monosaccharides from Poly G degradation appeared only after 2 h (Figure 3c). Overall, MhAly6 generated identical types of products from all three substrates.

After 24 h of reaction, the final products of sodium alginate degradation by MhAly6 were analyzed via electrospray ionization mass spectrometry (ESI-MS) (Figure 3d). The signals at *m/z* 193.03, 351.06, 527.09, and 703.12 corresponded to saturated monosaccharides, unsaturated disaccharides (DP 2), trisaccharides (DP 3), and tetrasaccharides (DP 4), respectively. The peaks at *m/z* 923.12 and 1056.19 represented oligosaccharides with DP 5–7, including one or two Na⁺ adducts. As listed in Table 1, similar to other alginate lyases of the PL6 family, MhAly6 degraded sodium alginate in an exolytic mode, producing AOSs with DP 2–7. Notably, MhAly6 is among the few alginate lyases capable of generating DP 7 oligosaccharides. These results highlight its strong potential for efficient sodium alginate degradation.

However, the heterogeneous mixture of high-DP products remains a significant challenge in AOS production using alginate lyases. In future studies, we plan to engineer MhAly6 through rational design and enzyme fusion technologies to enable the production of specific or single-DP AOSs, thereby reducing downstream separation and purification costs.

### 2.4. Structural Modeling and Evaluation of MhAly6

**Figure 4:**
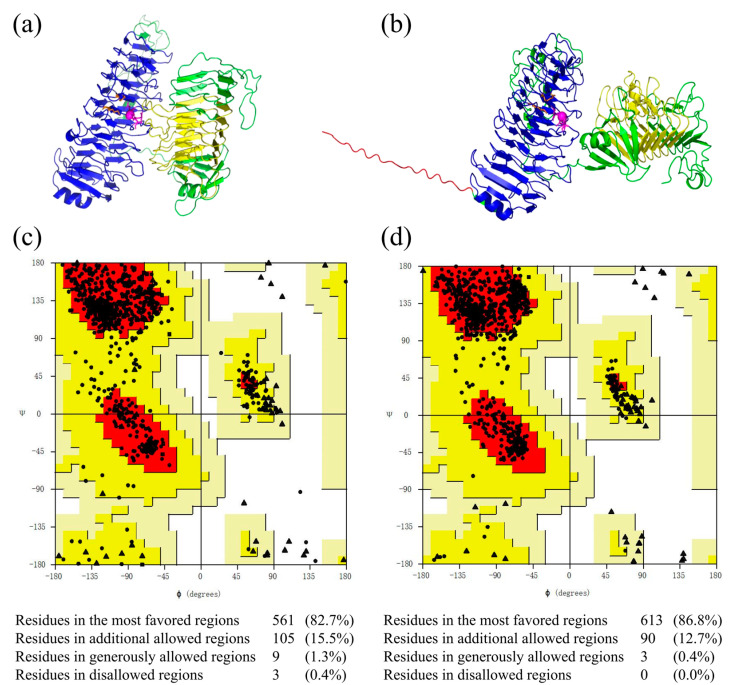
Structural modeling and evaluation of MhAly6. (**a**) Homology model generated by SWISS-MODEL. (**b**) The AlphaFold 3-predicted model. The signal peptide fragment is depicted in red, the PL6 family domain in blue (where Ca^2+^ and its binding sites are marked in purple, and PL6 family catalytic residues are marked in orange), the GH28 family domain in yellow, and the remaining structure in green. (**c**) Ramachandran plot analysis of the homology model. (**d**) Ramachandran plot analysis of the AlphaFold 3-based model. The color gradient from dark to light in each section represents the most favored regions, additional allowed regions, generously allowed regions, and disallowed regions, respectively. Triangles, squares, and dots indicate amino acids located in different regions.

To predict the three-dimensional structure of MhAly6 with high precision, homology modeling using the SWISS-MODEL server and AlphaFold 3 were performed simultaneously. On the SWISS-MODEL server, AlyGC (PDB ID: 5gkd), which shares 53.23% sequence identity with MhAly6, was selected as the template to generate a homology model of MhAly6 (Figure 4a), and the Global Model Quality Estimation (GMQE) and Qualitative Model Energy Analysis (QMEAN) scores provided by the server were used for evaluation. According to previous studies, models are considered valid when the GMQE and QMEAN scores fall within the ranges of 0–1 [47] and −4–0 [48], respectively. In this study, the homology model achieved GMQE and QMEAN values of 0.65 and −1.91, both meeting the criteria for validity. AlphaFold 3, a third-generation protein structure prediction model developed by DeepMind, represents a milestone in structural biology [49]. Benefiting from its advantages, we also employed AlphaFold 3 for modeling (Figure 4b). The results showed that the model predicted by AlphaFold 3 not only included the PL6 and GH28 family domains but also successfully captured the region of the signal peptide, which was absent in the homology model. Notably, the two methods yielded substantially different conformations, particularly in the signal peptide and C-terminal domain (Pro550-Tyr797). The SWISS-MODEL server constructs three-dimensional structures by aligning the target sequence with known templates based on homology. This method performs well when high-homology templates are available but may lead to inaccurate predictions in the absence of suitable templates. In contrast, AlphaFold3 builds three-dimensional structures by predicting the distances and angular relationships between amino acid residues. It is capable of generating high-quality predictions even without high-homology templates. Therefore, to obtain reliable molecular docking results, it is necessary to perform a Ramachandran plot analysis on both models.

The accuracy of both structures was further assessed using Ramachandran plot analysis via SAVES v6.0 (Figure 4c,d). According to the evaluation standards, models with more than 90% of residues in the most favored regions are considered high-quality, while those with more than 80% are regarded as reliable [50]. The homology model showed 82.70% of the residues in the most favored regions, while the AlphaFold 3-predicted model reported this proportion as 86.80%. Therefore, both models in this study were reliable. Notably, in the homology model, three residues were located in the disallowed regions, whereas the AlphaFold 3 model exhibited none. This clearly indicates the superior accuracy of the AlphaFold 3-predicted model. Although both models are valuable for future research, the structurally more complete AlphaFold 3-predicted model was selected for subsequent molecular docking studies between MhAly6 and DP 6 AOS.

**Figure 5:**
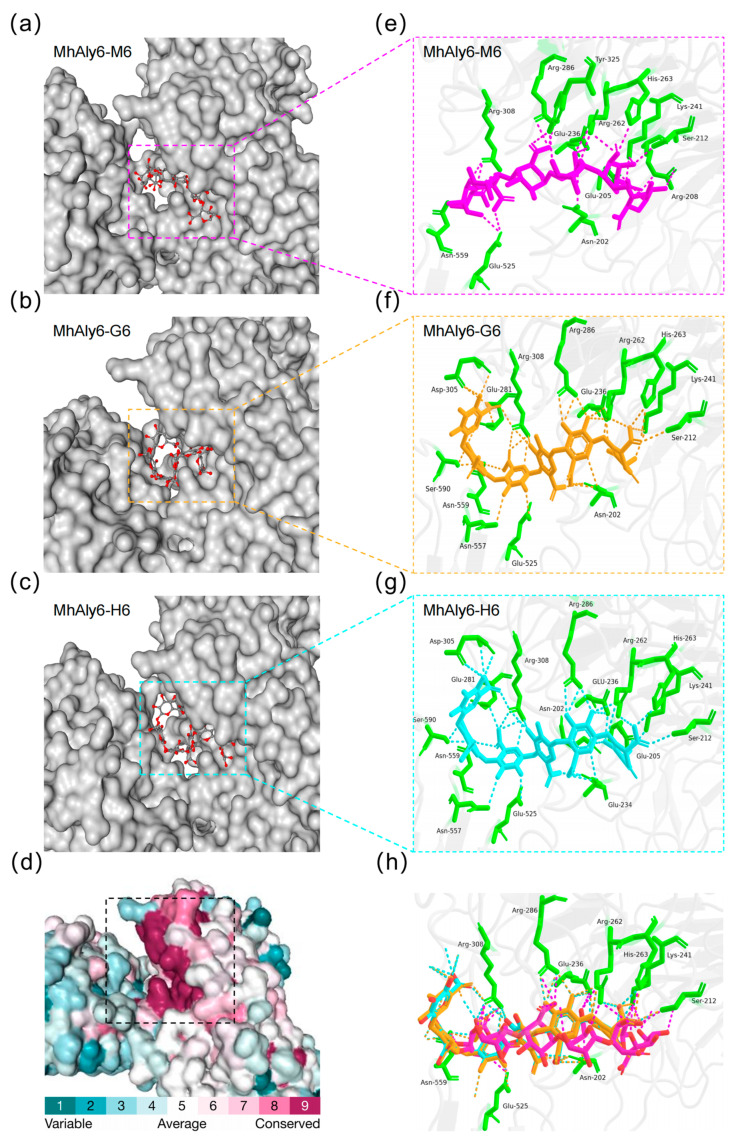
Substrate-binding groove, conservation analysis, and molecular docking results of MhAly6. (**a**) Binding positions of M6 in MhAly6. (**b**) Binding positions of G6 in MhAly6. (**c**) Binding positions of H6 in MhAly6. (**d**) Evolutionary conservation analysis of MhAly6. Amino acid residues were analyzed using the ConSurf program, with conservation grades color-coded on a gradient from most variable (turquoise) to most conserved (maroon). (**e**) Key residues interacting with M6 in MhAly6. (**f**) Key residues interacting with G6 in MhAly6. (**g**) Key residues interacting with H6 in MhAly6. (**h**) Key residues interacting with three substrates (M6, G6, and H6) in MhAly6. Key residues are shown in green; M6, G6, and H6 molecules are depicted in purple, yellow, and blue, respectively. Conserved catalytic sites (Lys241/Arg262) and Ca^2+^ binding sites (Asn202/Glu234/Glu236).

In order to identify the key substrate recognition sites of MhAly6, this study screened the molecular docking results with the lowest binding free energy for analysis. The results indicated that M6, G6, and H6 could bind to the cleft of the PL6 family domain of MhAly6 (Figure 5a–c), and this region was highly conserved (Figure 5d). Interestingly, although the substrate binding sites of M6, G6, and H6 were not exactly the same, the binding sites of G6 and H6 showed considerable similarity. Further examination of the binding interactions between the three substrates and MhAly6 revealed that in the MhAly6-M6 complex, Asn202, Glu205, Arg208, Ser212, Glu236, Lys241, Arg262, His263, Arg286, Arg308, Tyr325, Glu525, and Asn559 could form hydrogen bonds with the M6 molecule (Figure 5e). In the MhAly6-G6 complex, Asn202, Ser212, Glu236, Arg262, Lys241, His263, Glu281, Arg286, Asp305, Arg308, Glu525, Asn557, Asn559, and Ser590 could form hydrogen bonds with the G6 molecule (Figure 5f). In the MhAly6-H6 complex, Asn202, Glu205, Ser212, Glu234, Glu236, Lys241, Arg262, His263, Glu281, Arg286, Asp305, Arg308, Glu525, Asn557, Asn559, and Ser590 could form hydrogen bonds with the H6 molecule (Figure 5g). It is worth noting that ten residues—Asn202, Ser212, Glu236, Lys241, Arg262, His263, Arg286, Arg308, Glu525, and Asn559—could form hydrogen bonds with all three substrates in MhAly6 (Figure 5h). In the docking results of AlyRm1 with the tetrasaccharides (M4 and G4), among the nine residues that can form hydrogen bonds with both substrates, five are identical to those in MhAly6 (Glu201, Ser211, Glu235, Lys240, and Arg261 in AlyRm1 correspond to Glu202, Ser212, Glu236, Lys241, and Arg262 in MhAly6, respectively).. This indicates that AlyRm1 and MhAly6 share certain similarities in their substrate-binding grooves [45]. Specifically, Asn202 and Glu236 served as the binding sites for Ca^2+^, while Lys241 and Arg262 were identified as the key catalytic residues. In the study by Xu et al., alanine substitution of the catalytic residues Lys220 and Arg241 in the *G. chathamensis* S18K6 alginate lyase AlyGC (corresponding to Lys241 and Arg262 in MhAly6) led to mutants that lost nearly 95% of the wild-type enzyme’s activity toward Poly G [33]. Interestingly, Glu525 is located in the linker between the PL6 family domain and the GH28 family domain, while Asn559 resides within the GH28 family domain. This indicates that both the linker and the GH28 family domain participate in substrate binding in MhAly6. In Zheng et al.’s study, a similar phenomenon was observed. The alginate lyase AlyRm1 from *R. marina*, which they characterized, contains a PL6 domain (Val24-Gly396) and an FlgD-Ig-like domain (Phe501-Ala562). In this enzyme, Glu543 located within the FlgD-Ig-like domain forms hydrogen bonds with G4 molecules, facilitating substrate binding [45]. This suggests that non-catalytic domains (such as other polysaccharide catalytic domains, carbohydrate-binding modules, or other unverified functional structures) have an undeniable effect on substrate binding by the catalytic domains in mature alginate lyases. This finding also reminds us of the importance of considering the effects of non-catalytic domains when conducting truncation studies on high-molecular-weight alginate lyases, as these domains may significantly affect the enzyme function.

### 2.5. Antioxidant Activity of AOSs Derived from MhAly6-Mediated Degradation

AOSs derived from alginate degradation have attracted significant attention due to their exceptional antioxidant properties, with applications in food industries and pharmaceutical development. In this study, the antioxidant activity of AOSs generated by MhAly6-mediated sodium alginate degradation was assessed using multiple assays, including ferric reducing power and the scavenging of hydroxyl, DPPH, and ABTS radicals.

**Figure 6:**
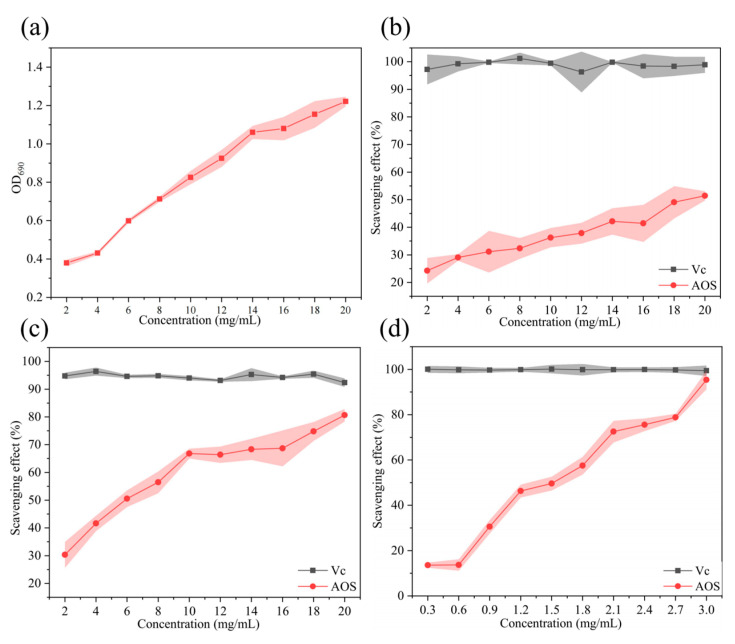
Antioxidant functions of AOSs produced by MhAly6. (**a**) The ferric reducing ability of AOSs. (**b**) Hydroxyl radical scavenging activity of AOSs. (**c**) DPPH radical scavenging activity of AOSs. (**d**) ABTS radical scavenging activity of AOSs. (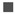: Vc, 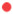: AOS).

Antioxidant activity is closely linked to the reducing capacity, making the ferric reducing power a common metric for assessing antioxidants. As shown in Figure 6a, the reducing power of the AOSs at 690 nm increased from 0.37 to 1.22 in a concentration-dependent manner. Reductones are a class of carbonyl-containing organic compounds with strong reducing properties. Their structural characteristics enable them to neutralize hydroxyl radicals by donating hydrogen atoms, thereby interrupting oxidative chain reactions. The presence of reductone groups in AOS molecules confers electron-donating characteristics, allowing them to reduce high-valent metal ions (e.g., Fe^3^⁺→Fe^2^⁺) while scavenging radicals.

The hydroxyl radical is a highly reactive oxygen species with potent oxidizing capacity. In biological systems, hydroxyl radicals can attack deoxyribose and bases in DNA, leading to base modifications and strand breaks, ultimately resulting in gene mutations or apoptosis. As shown in Figure 6b, although the hydroxyl radical scavenging efficacy of AOSs was less potent than that of ascorbic acid (Vc), its activity increased progressively with concentration. At 20 mg/mL, the AOSs achieved a hydroxyl radical scavenging rate of 51.41 ± 1.68% (Figure 6b). Figure 6c shows that the same concentration (20 mg/mL) of AOSs exhibited superior DPPH radical scavenging activity (80.64 ± 1.40%), surpassing the results reported previously [51]. Remarkably, in Figure 6d, the AOSs at 3 mg/mL displayed an extraordinary ABTS radical scavenging rate of 95.39 ± 4.10%, a phenomenon not observed in hydroxyl radical or DPPH radical scavenging experiments. According to Kelishomi et al., the antioxidant mechanism of AOSs may involve hydrogen atom transfer and radical adduct formation, with the formation of double bonds between C-4 and C-5 playing a crucial role in these interactions [52].

These findings collectively indicated that AOSs derived from MhAly6-mediated alginate degradation possess significant potential as a natural antioxidant for applications in food preservation, cosmetics, and nutraceuticals.

## 3. Materials and Methods

### 3.1. Chemicals and Strains

Sodium alginate (viscosity: 200 ± 20 mPa·s, purity ≥ 98%) was purchased from Aladdin Biochemical Technology Co., Ltd. (Shanghai, China). Poly M (M content: 92.6%, G content: 7.4%) and Poly G (G content: 86.2%, M content: 13.8%) were obtained from Qingdao BZ Oligo Biotech Co., Ltd. (Qingdao, China). Kanamycin and isopropyl-*β*-D-thiogalactopyranoside (IPTG) were acquired from Solarbio Science & Technology Co., Ltd. (Beijing, China). Other chemicals used in this study were of analytical grade. TLC silica gel plates (60F 254) were purchased from Merck Group (Darmstadt, Germany). Primers for MhAly6 gene cloning were synthesized by Sangon Biotech Co., Ltd. (Shanghai, China). *E. coli* DH5α was used for plasmid construction, and *E. coli* BL21(DE3) was employed for protein expression.

### 3.2. Bioinformatics Analysis

The genome of *M*. *hitae* R32 strain (GenBank ID: GCA008692195) was annotated for alginate lyases using the dbCAN2 database (https://bcb.unl.edu/dbCAN2/blast.php, accessed on 12 January 2025). The signal peptide of MhAly6 was predicted using the SignalP 5.0 online tool (https://services.healthtech.dtu.dk/services/SignalP-5.0, accessed on 12 January 2025). The protein domains of MhAly6 were analyzed via the NCBI Conserved Domain Database (https://www.ncbi.nlm.nih.gov/Structure/cdd/wrpsb.cgi, accessed on 12 January 2025). A phylogenetic tree of the PL6 subfamily alginate lyases was constructed using MEGA 11.0 software, based on the maximum likelihood method with 1000 bootstrap replicates. The multiple sequence alignment results were visualized using ESPript 3.0 (http://espript.ibcp.fr/ESPript/ESPript/, accessed on 21 January 2025). The Mw, isoelectric point, and amino acid composition of MhAly6 were analyzed using the Expasy-ProtParam tool (https://web.expasy.org/protparam accessed on 21 January 2025). The solubility expression potential of this enzyme in *E. coli* was predicted using SoluProt (https://loschmidt.chemi.muni.cz/soluprot, accessed on 25 January 2025).

### 3.3. Cloning, Expression and Purification of MhAly6

The MhAly6 gene was amplified from the genomic DNA of *M*. *hitae* R32 using the forward primer (MhAly6-F: 5′-GGATCTTCCAGAGATTCTAGAATGCAAAAACAACTGGTGGAGAA-3′) and the reverse primer (MhAly6-R:5′-CTGCCGTTCGACGATAAGCTTTTAATAACTTAATAGTCCTATGGTTTCTATACC-3′). The amplified fragment was then digested with *Xba*I and *Hin*dIII restriction enzymes and ligated into the pET28a(+) vector between the *Xba*I and *Hin*dIII restriction sites. The ligation product was subsequently transformed into *E. coli* DH5α competent cells. The construct was sequenced by CWBIO Biotechnology Co., Ltd. (Taizhou, China) for verification. After confirmation, the plasmid was transformed into *E. coli* BL21(DE3) competent cells.

The alginate lyase MhAly6 was expressed in *E. coli* BL21(DE3). Briefly, the recombinant *E. coli* BL21(DE3) strain harboring the MhAly6 gene was inoculated at (2% *v*/*v*) into LB broth containing kanamycin (100 mg/L) and cultured at 37 °C with shaking at 150 rpm until the OD_600_ reached approximately 0.5. Protein expression was induced by adding IPTG to a final concentration of 0.1 mM. Following induction, the culture was incubated at 16 °C with shaking at 150 rpm for 16 h to produce large quantities of the recombinant protein. Cells were harvested by centrifugation, resuspended in 20 mM Tris-HCl buffer (pH 7.4), and lysed by ultrasonication. The recombinant protein was purified using a His-tag Protein Purification Kit (Beyotime Biotechnology Co., Ltd., Shanghai, China) with a linear imidazole gradient (20–250 mM). Protein purity was analyzed by 12% SDS-PAGE, and the eluted protein was concentrated via ultrafiltration. Protein concentration was determined using a Bradford Protein Assay Kit (Beyotime Biotechnology Co., Ltd., Shanghai, China).

### 3.4. Biochemical Characterization of MhAly6

The alginate lyase activity of MhAly6 was measured using the DNS method [53]. Briefly, 900 μL of 0.5 mg/mL sodium alginate was mixed with 100 μL of the diluted enzyme solution (0.25 mg/mL, the enzyme concentration in all subsequent steps was 0.25 mg/mL) and incubated at 40 °C for 10 min. The reaction was terminated by adding 1.0 mL DNS, followed by boiling at 100 °C for 5 min to develop color. After cooling to room temperature (25 °C ± 3 °C), the solution was diluted to a final volume of 5.0 mL, and the absorbance was measured at 540 nm. Heat-inactivated enzyme was used as the control. One unit (U) of the enzyme activity was defined as the amount of enzyme required to release 1 μg of reducing sugar (glucose equivalent) per min.

The optimal temperature for MhAly6 activity was determined by measuring enzyme activity at various temperatures ranging from 25 °C to 65 °C in 20 mM glycine-NaOH buffer (pH 8.0). Reactions were terminated by boiling after 20 min of incubation. Thermal stability was evaluated by pre-incubating the enzyme at 40 °C, 45 °C, or 50 °C for 0–210 min. The optimal pH was determined using buffers with pH values ranging from 3.0 to 10.0 (sodium citrate buffer for pH 3.0–6.0, sodium phosphate buffer for pH 6.0–8.0, and glycine-NaOH buffer for pH 8.0–10.0), Tris-HCl buffer (pH 8.0) was used as 100%. The effect of NaCl on enzyme activity was evaluated by measuring activity in the presence of 0–1.0 M NaCl. Substrate specificity was determined using sodium alginate, Poly M, and Poly G as substrates. The influence of metal ions and chemical reagents on MhAly6 was investigated by adding them to the reaction mixture at final concentrations of 1, 5, or 10 mM (for ions) or 0.1%, 0.5%, and 1% (*w*/*v* for reagents).

### 3.5. Analysis of Degradation Products

The purified enzyme was mixed separately with three substrates—sodium alginate, Poly M, and Poly G—at a 1:9 ratio and allowed to react under optimal conditions for 0.5–24 h. To terminate the reactions, samples were boiled in a water bath for 10 min. The resulting products were purified via ethanol precipitation. The procedure involved first centrifugation at 10,000 rpm for 10 min to remove inactivated MhAly6 and undegraded substrate. The supernatant was then collected, and anhydrous ethanol was added to a final concentration of 70%. The mixture was incubated at 4 °C for 24 h to allow precipitation. After precipitation, the mixture was centrifuged again at 10,000 rpm for 10 min to remove the precipitate. The supernatant was then concentrated by rotary evaporation at 60 °C to obtain the AOS samples. After purification, the AOSs were dissolved in deionized water to prepare a 10 mg/mL solution, sterilized by filtration through a 0.22 μm sterile membrane filter, and stored at 4 °C for subsequent use. The product distribution at different degradation times was initially analyzed by TLC. The sample was loaded at a volume of 5 μL, and a developing solvent system consisting of n-butanol–formic acid–water = 4:6:1 (*v*/*v*/*v*) was used. Visualization was performed with a 10% sulfuric acid in ethanol solution as the staining reagent, and coloration was carried out by heating at 105 °C for 5 min. To further characterize the products, negative-ion ESI-MS (Thermo Fisher Scientific Q Exactive Focus, Waltham, MA, USA) was employed to determine the distribution and DP of the products obtained after 24 h of degradation using sodium alginate as the substrate. Instrument settings included a spray voltage of 3 kV, sheath gas flow rate of 35 arb, auxiliary gas flow rate of 10 arb, and a resolution of 70,000 FWHM, with deionized water as the solvent.

### 3.6. Structural Modeling and Molecular Docking of MhAly6

The three-dimensional structure of MhAly6 was predicted using both the SWISS-MODEL server and AlphaFold 3. The resulting models were evaluated using the SAVES v6.0 server. Molecular docking of MhAly6 with AOSs (DP 6) was performed using AutoDock-Vina 1.1.2. The ligand Poly M (M6, CID: 71761996) was downloaded from PubChem (https://pubchem.ncbi.nlm.nih.gov/, accessed on 3 February 2025). Poly G (G6) and Poly MG (H6, a hexasaccharide formed by alternating *β*-1,4-glycosidic bonds between mannuronic and guluronic acids) were generated using KingDraw 3.0. Format conversions were carried out using Open Babel 3.1.1. The docking mode with the lowest binding energy was selected for further analysis. Conservation analysis was performed using Consurf, and the docking results were visualized using PyMOL 3.1.

### 3.7. Antioxidant Properties of the Alginate Degradation Products Produced by MhAly6

#### 3.7.1. Ferric Reducing Capacity

The ferric reducing capacity of the alginate degradation products was determined according to a previously described method with minor modifications [54]. Briefly, 50 μL of degradation product solutions with different concentrations (2–20 mg/mL) were added to a 96-well plate, followed by sequential addition of 50 μL sodium phosphate buffer (pH 6.6, 20 mM) and 50 μL of potassium ferricyanide solution (1% *w*/*v*). After incubation at 50 °C for 20 min, 50 μL of (10% *w*/*v*) trichloroacetic acid containing (1% *w*/*v*) ferric chloride was added to the reaction mixture and kept at room temperature (25 °C ± 3 °C) for 10 min. The absorbance of the mixture was measured at 690 nm.

#### 3.7.2. Hydroxyl Radical Scavenging Capacity

The hydroxyl radical scavenging capacity of the degradation products was determined using a modified salicylic acid method [55]. Briefly, 50 μL of the degradation product solutions at different concentrations (2–20 mg/mL), 50 μL of FeSO_4_ solution (9 mM), 50 μL of salicylic acid–ethanol solution (9 mM), and 50 μL of H_2_O_2_ (8.8 mM) were sequentially added to a 96-well plate. After thorough mixing, the mixture was incubated in a 37 °C water bath for 30 min, and the absorbance at 510 nm was recorded as A. A blank control (absorbance B) was prepared by replacing H_2_O_2_ with deionized water, and a negative control (absorbance C) was prepared by replacing the degradation product solution with deionized water. Vc at the same concentrations was used as a positive control. The hydroxyl radical scavenging rate was calculated using Formula (1):

Scavenging effect (%) = (B − A)/(B − C) × 100(1)

#### 3.7.3. Scavenging Activity of 2,2-Diphenyl-1-picrylhydrazyl (DPPH)

Under dark conditions, different concentrations (2–20 mg/mL) of degradation product solutions and an equal volume of DPPH solution (0.2 mM) were sequentially added to a 96-well plate. After incubation at 25 °C for 30 min, the absorbance at 517 nm was measured and recorded as A [56]. A blank control (absorbance B) was prepared by replacing the DPPH solution with absolute ethanol, and a negative control (absorbance C) was prepared by replacing the degradation product solution with deionized water. Vc at the same concentrations was used as a positive control. The DPPH radical scavenging rate was calculated using Formula (1).

#### 3.7.4. Scavenging Activity of 2,2′-Azinobis-(3-ethylbenzthiazoline-6-sulphonate) (ABTS)

The ABTS free radical scavenging activity was determined using a modified version of a previously described method [57]. The stock solution of ABTS radical was prepared by mixing ABTS (7 mM) and K_2_S_2_O_8_ (88 mM) solutions at a volume ratio of 5:88, followed by storage in the dark for 16 h to generate ABTS free radicals. The stock solution was diluted with absolute ethanol to achieve an absorbance of 1.4 ± 0.05 at 405 nm, yielding the ABTS radical working solution. Equal volumes of the ABTS radical working solution and degradation product solutions at different concentrations (0.3–3.0 mg/mL) were mixed and incubated at 25 °C for 30 min. The absorbance at 405 nm was recorded as A. A blank control (absorbance B) was prepared by replacing the ABTS radical working solution with absolute ethanol, and a negative control (absorbance C) was prepared by replacing the degradation product solution with deionized water. V_C_ at the same concentrations was used as a positive control. The ABTS radical scavenging rate was calculated using Formula (1).

## 4. Conclusions

To date, no alginate lyases from the genus *Mesonia* have been characterized. This study represents the first report on the biochemical properties of an alginate lyase from this genus, providing critical insights for understanding and utilizing the biotechnological potential of the *Mesonia* species. In this paper, we described the cloning and characterization of a novel PL6 family alginate lyase, MhAly6, from *M. hitae* R32. The MhAly6 exhibited optimal activity at 45 °C and pH 9.0, along with exceptional thermal stability at 40 °C, retaining over 50% activity after 210 min of incubation. The enzyme showed tolerance to multiple metal ions at 1 mM and displayed Na⁺-dependent activation. MhAly6 efficiently degraded sodium alginate, Poly M, and Poly G, with the highest activity towards its natural substrate sodium alginate. The degradation products were AOSs with DPs ranging from 2 to 7. The antioxidant assays revealed that MhAly6-derived AOSs displayed significant radical-scavenging activity, emphasizing their potential for applications in antioxidant-based products. In our ongoing research, we plan to perform site-directed mutagenesis to replace Lys498 with Asp, and re-evaluate its pectin degradation capability. Additionally, we are engineering MhAly6 by strategically increasing the proportions of Pro and Arg residues, with the goal of optimizing its enzymatic activity and thermostability under high-temperature conditions.

## Figures and Tables

**Table 1:** Characteristics of the PL6 family alginate lyases from different sources: a recent overview.

Enzyme	Source	Substrate Specificity ^a^	AOS Products (DP)	Action Mode	Activators ^b^	Inhibitors ^c^	Optimal Temperature (°C)/pH	Stability	Reference
MhAly6	*M. hitae* R32	SA > PM > PG	2–7	Exo	Ni^2+^ (5 mM); Cu^2+^ (1 mM); Ca^2+^ (1 mM)	Cu^2+^ (5 mM); EDTA (10 mM); Fe^3+^ (10 mM)	45/9.0	40 °C,60 min, remaining 80%	This study
TsAly6A	*Thalassomonas* sp. LD5	PG > SA > PM	2–3	Endo	Ca^2+^ (-); Mg^2+^ (-); Fe^3+^ (-)	EDTA (-); SDS (-); Na^+^ (500 mM)	35/8.0	40 °C, 60 min, remaining 29%	[38]
ALFA4	*Formosa algae* KMM 3553^T^	-	2 (Main)	-	-	-	30/8.0	37 °C, 100 min, remaining 50%	[39]
AlyPL6	*Pedobacter hainanensis* NJ-02	SA > PG > PM	2–6	Exo	Na^+^ (1 mM)	Mn^2+^ (1 mM); Zn^2+^ (1 mM); Co^2+^ (1 mM)	45/10	40 °C, 60 min, remaining 50%	[40]
OUC-ScCD6	*Streptomyces ecolicolor* A3(2)	SA > PM > PG	2–10	Endo	Mn^2+^ (1 mM); Fe^3+^ (1 mM); Zn^2+^ (1 mM)	EDTA(1 mM); Ni^2+^ (1 mM); Cu^2+^ (1 mM)	50/9.0	40 °C, 60 min, remaining 50%	[41]
AlyM2	*Pseudoalteromonasarctica* M9	PMG > SA > PM > PG	3 (Main)	-	-	-	30/8.0	-	[42]
AlgL6	*Microbulbifer* sp. ALW1	PG > SA > PM	2–4	Exo	Tween 80(1%); Tween 20(1%); Na^+^ (500 mM)	Cu^2+^ (10 mM); Fe^2+^ (10 mM); Ba^2+^ (10 mM)	35/8.0	40 °C, 60 min, remaining 19%	[43]
VpAly-VII	*Vibrio pelagius* WXL662	SA > PM > PG	3–6	Endo	-	-	50/8.0	-	[44]
AlyRm1	*Rubrivirga marina*	PM > SA > PG	2–5	Exo	SDS(1 mM); Ca^2+^ (1 mM); K^+^ (1 mM)	EDTA(1 mM); Zn^2+^ (1 mM); Mn^2+^ (1 mM)	30/10.0	40 °C, 60 min, remaining 20%	[45]
TAPL6	*Thalassotaleaalgicola*	PM > SA > PG	2–6	Exo	Mg^2+^ (1 mM); Ca^2+^ (1 mM); K^+^ (1 mM)	Zn^2+^ (1 mM); Fe^2+^ (1 mM); Ni^2+^ (1 mM)	25/10.0	40 °C, 60 min, remaining 20%	[46]
AlyRmA	*Rhodothermus marinus*	SA > PM > PG	2–4	Exo	Mg^2+^ (1 mM); Ca^2+^ (1 mM)	Cu^2+^ (1 mM); Zn^2+^ (1 mM); Ni^2+^ (1 mM)	70/8.0	70 °C, 60 min, remaining 100%	[11]

^a^: SA: sodium alginate; PM: Poly M; PG: Poly G; PMG: Poly MG; -: not determined; ^b^: Metal ions or chemical reagents that increased the enzyme activity under a certain concentration (the concentration); ^c^: Metal ions or chemical reagents that suppressed enzyme activity under a certain concentration (the concentration).

## Data Availability

The original contributions presented in this study are included in the article/Supplementary Material. Further inquiries can be directed to the corresponding authors..

## Supplementary Materials

The following supporting information can be downloaded at https://www.mdpi.com/article/10.3390/md23040176/s1: Figure S1: Multiple sequence alignment analysis of MhAly6 with selected polygalacturonases of the GH28 family (partial sequences). Catalytic sites are marked with red circles.

## Abbreviations

M	*β*-D-mannuronic acid
G	α-L-guluronic acid
Mw	Molecular weight
Poly M	Poly *β*-D-mannuronic acid
Poly G	Poly α-L-guluronic acid
AOS	Alginate oligosaccharides
DP	Degree of polymerization
PL	Polysaccharide lyase
IPTG	Isopro-pyl-*β*-D-thiogalactopyranoside
TLC	Thin-layer chromatography
ESI-MS	Electrospray ionization mass spectrometry
Vc	Ascorbic acid
DPPH	2,2-Diphenyl-1-picrylhydrazyl
ABTS	2,2′-Azinobis-(3-ethylbenzthiazoline-6-sulphonate)
