# Supplementary material for: The Identification and Characterization of a Novel Alginate Lyase from Mesonia hitae R32 Exhibiting High Thermal Stability and Potent Antioxidant Oligosaccharide Production
Source: Mar Drugs. 2025 Apr 17;23(4):176. doi: 10.3390/md23040176 (PMC12028748; doi:10.3390/md23040176)
Supplement: Supplementary file 1 [file marinedrugs-23-00176-s001.zip › marinedrugs-3557056-supplementary.pdf]

Supplementary Materials

IThe Identification and Characterization of a Novel Alginate Lyase from *Mesonia hitae* R32 Exhibiting High Thermal Stability and Potent Antioxidant Oligosaccharide Production

Yongshang Ye, Zhiyu Li, Ying Zhou *, Xiujun Gao * and Dingfan Yan

Department of Biotechnology, School of Marine Science and Technology, Harbin Institute of Technology, Weihai, 264209, China
*****zhou.ying@hit.edu.cn(Y. Z.); gaoxiujun@hitwh.edu.cn(X. J. G.)

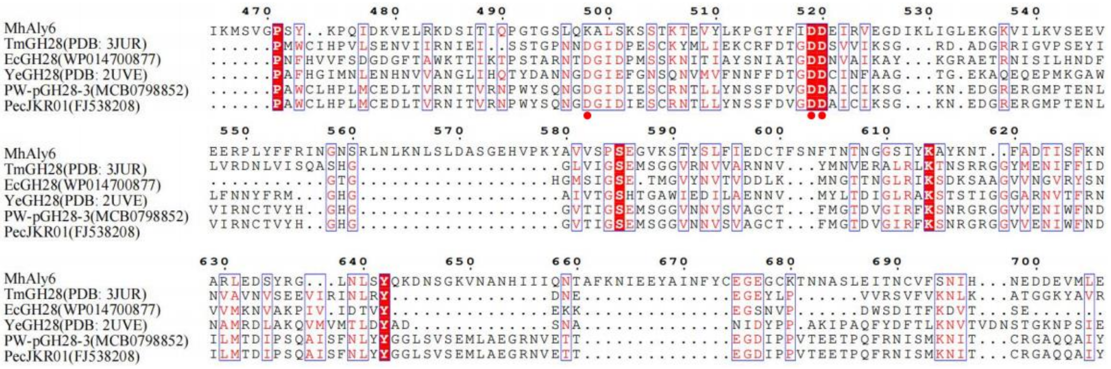

Figure S1. Multiple sequence alignment analysis of MhAly6 with selected polygalacturonases of the GH28 family (partial sequences). Catalytic sites are marked with red circles.
